# Antimicrobial Activity of Lactones

**DOI:** 10.3390/antibiotics11101327

**Published:** 2022-09-29

**Authors:** Marcelina Mazur, Dorota Masłowiec

**Affiliations:** 1 Department of Food Chemistry and Biocatalysis, Wrocław University of Environmental and Life Sciences, Norwida 25, 50-375 Wrocław, Poland; 2 Institute of Health Sciences, University of Opole, Katowicka 68, 45-060 Opole, Poland

**Keywords:** lactones, antimicrobial compounds, antibacterial activity, isolation of lactones, quorum sensing

## Abstract

The development of bacterial resistance to antibiotics and the consequent lack of effective therapy is one of the biggest problems in modern medicine. A consequence of these processes is an urgent need to continuously design and develop novel antimicrobial agents. Among the compounds showing antimicrobial potential, lactones are a group to explore. For centuries, their antimicrobial activities have been used in folk medicine. Currently, novel lactone compounds are continuously described in the literature. Some of those structures exhibit high antimicrobial potential and some are an inspiration for design and synthesis of future drugs. This paper describes recent developments on antimicrobial lactones with smaller ring sizes, up to seven membered ε-lactones. Their isolation from natural sources, chemical synthesis, synergistic activity with antibiotics, and effects on quorum sensing are presented herein.

## 1. Introduction

**Figure 1:**
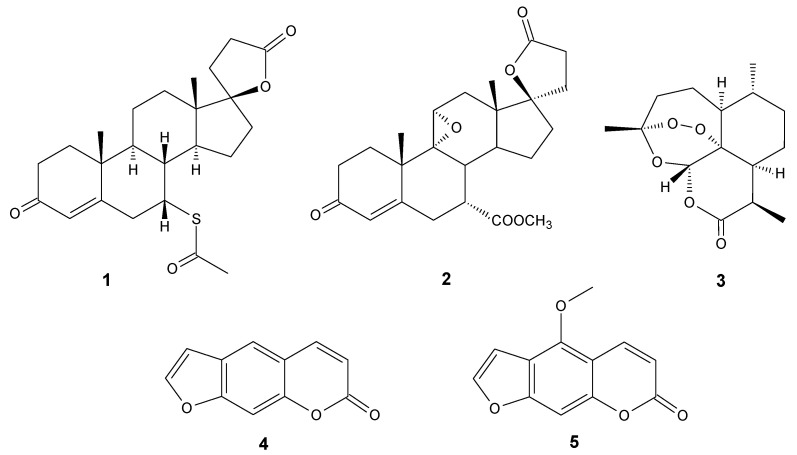
Spironolactone (**1)**, eplerenone (**2**), artemisinin (**3**), psoralen (**4)**, bergaptene (**5**).

Lactones are a group of compounds widely distributed in nature [1,2,3,4]. Chemically, they can be classified as intramolecular esters of hydroxycarboxylic acids with different ring sizes. Due to the stability of the ring structure, the most common are γ- and δ-lactones with five- and six-membered rings [5]. However, lactones with other ring sizes can also be isolated from natural sources or obtained by chemical synthesis [6,7]. Compounds with the lactone moiety may have completely different carbon skeletons and may be assigned to different classes of compounds, such as sesquiterpene lactones [8,9], coumarins [10], or steroid skeleton lactones [11]. Thanks to this diversity, it is an extremely interesting group that exhibits several important biological properties. Among these activities, the most frequently mentioned are cytotoxic [12,13,14], anti-inflammatory [15,16], antiplasmodial [17], antiviral [18], and antibacterial. Therefore, a wide selection of articles provides an overview of those biological properties or most often presents, in detail, the anticancer properties [4,5,9,19]. However, the topic of the antimicrobial activity of this group of compounds deserves more attention. For that reason, the goal of this work was to present some recent developments in the area of lactone compounds with antimicrobial activity in context of the constant search for new therapeutic agents. The World Health Organization scored this area as one of the main global concerns [20]. It should be noted that the emergence of strains resistant to antibiotics is associated with many threats to human and animal life and health. Considering that, at present, the new compounds used in antimicrobial therapy are modifications of already known antibiotics and their mechanism of action does not differ from their precursors, microorganisms develop resistance to these substances extremely quickly [21,22]. The widespread use and overuse of antibiotics also contributes to this problem. Therefore, the search for new substances that do not belong to the already known groups of antibiotics is of particular importance. Lactones may be a target group here, due to them having such a wide potential as biologically active compounds. So far, they have been used as drugs for hypertension (spironolactone **1**, eplerenone **2**), malaria (artemisinin **3**), vitiligo (psoralen **4** and bergaptene **5**), and also as antibiotics belonging to macrolides (Figure 1) [5]. However, as macrolides are a wide topic itself [23], we did not cover it in this review; instead, we focused mainly on lactones with small ring sizes, up to seven-membered ε-lactones.

## 2. Isolation

### 2.1. Lactones Isolated from Plants

From the beginning, drug development has been correlated with the use of various medicinal plants and remedies from other natural sources [24]. Nowadays, we link the spectrum of biological activity with specific compounds isolated from these plants and microorganisms. After centuries of their application in traditional folk medicines, we are still able to discover new active substances responsible for their therapeutic properties. Among them, lactones play an important role. Some of those compounds are well-known as one of the plant’s chemical defense elements against microbial pathogens. One of the most distinguished groups are sesquiterpene lactones, known for their broad-spectrum biological potential including antimicrobial properties [25,26]. The mechanism of their action is often related with the presence of α,β-unsaturated γ-lactone moiety, although other structural features can also play an important role. The occurrence of an α-methylene group or an α,β-unsaturated lactone ring provides an opportunity for alkylation by nucleophilic Michael addition and results in irreversible bonding with other molecules, for example, enzymes [19]. Interestingly, this type of mechanism is often related with the cytotoxic potential of this group of compounds. The antimicrobial activity of sesquiterpenes can also be associated with changes in protein synthesis, altered cellular permeability, or interaction with cell wall phospholipids disturbing the membrane integrity [9]. In recent years, several new antimicrobial sesquiterpene lactones were isolated. The sources of them are often medicinal plants, marine organisms, and fungi.

*Artemisia annua* L. (Asteraceae) is an annual herb native to Asia and has been used for many centuries in traditional Asian medicine for the treatment and prevention of fever and chills. Kim et al. [27] proposed antimicrobial activity of artemisinin (**3**) against periodontopathic microorganisms—Gram-negative anaerobic bacteria such as *Aggregatibacter actinomycetemcomitans*, *Fusobacterium* species, *Prevotella intermedia*. Periodontal disease and severe periodontitis can result in periodontal destruction, pain, alveolar bone resorption, and tooth loss. Appalasamy et al. [28] indicated that artemisinin can be an effective drug against not only Gram-negative *Salmonella* sp. but also Gram-positive *Staphylococcus aureus*, *Bacillus subtilis*. In another study by Boulanger et al. [29], helenalin (**6**) was presented as a strong antimicrobial agent in vivo against *S. aureus* in mouse models with infected mammary glands.

As with artemisinin (**3**), many other known sesquiterpene lactones, with a wide spectrum of biological activity, are currently under investigation for their antimicrobial properties. Costunolide (**7**) was first isolated from costus (*Saussurea lappa* Clarke) root and then isolated from various other plant species. Kim et al. reported that this compound, in addition to antioxidant, anti-inflammatory, antiallergic, bone remodeling, neuroprotective, anticancer, and antidiabetic properties, has antimicrobial properties against *Mycobacterium tuberculosis*, *Mycobacterium avium*, *Helicobacter pylori* [13]. The activities are expressed as minimum inhibitory concentration (MIC) and are presented in Table 1. Häkkinen at. al reported costunolide activity against *P. aeruginosa* IBRS P001 [26]. Another known sesquiterpene lactone—lactucopicrin (**8**), derived from the plant *Lactuca virosa* (wild lettuce), as well as being found in some related plants such as *Cichorium intybus*, exhibits antimicrobial activity against *S. aureus* ATCC 11632, *P. aeruginosa* ATCC 27853, *P. aeruginosa* IBRS P001. Parthenolide (**9**), a sesquiterpene lactone derived from the leaves of feverfew (*Tanacetum parthenium*), is considered a main bioactive component of this herb. Feverfew has been used orally or as an infusion for the treatment of migraine, arthritis, fever, and stomachache. Besides its anti-inflammatory and antimigraine properties, and anticancer in a variety of cell lines, parthenolide also shows antimicrobial activities against *S. aureus* strains and *P. aeruginosa* ATCC 27853. It contains an α-methylene-γ-lactone ring and an epoxide moiety which are able to interact with the nucleophilic groups of biologically important molecules [26,30]. 

*Anvillea garcinii* is a medicinal plant from the Asteraceae family, commonly known as Arabian oxeye. It is well-known for its medicinal properties and used for intestinal diseases, liver and lung disease, digestive problems, and as antidiabetic. In an ethanolic leaves extract, two new guaianolides and four sesquiterpene lactones were found. Their antimicrobial activity was evaluated against yeast *Candida parapsilosis* and *Candida albicans* but also several Gram-positive and Gram-negative strains of bacteria. The guaianolide-type lactones **10** and **11** were active antifungals and displayed remarkable antibacterial activity against *S. aureus* (MIC values of compound **10**—0.32 and compound **11**—1.4 µg/mL) and *Escherichia fergusonii* (MIC values of compound **10**—1.7 and compound **11**—3.5 µg/mL) [31]. 

A further example of Asteraceae is *Schkuhria pinnata* (Lam.) or Kuntze ex Thell, native to South America but also widespread as a weed in South Africa. Traditionally, it is used to treat bacterial infections and inflammations such as dermatitis, eczema, acne, eye infections, pneumonia, stomach problems, oedema, or even diabetes. For that reason, the search for compounds with antibacterial activity is particularly pertinent in this plant. From the plant’s extract, several sesquiterpene lactones were isolated and evaluated for their antibacterial potential. The most active was the mixture of two compounds **12** and **13** containing an α-methylene group in γ-lactone moiety. The lower MIC value 46.8 µg/mL was determined for *P. aeruginosa* among other tested bacteria such as *E. coli* (125 µg/mL), *E. faecalis* (125 µg/mL), or *S. aureus* (62.5 µg/mL). Ampicillin was used as the positive control with MIC values ranging from 20 to 80 µg/mL [32].

Moderate antibacterial activity was detected for two germacrane derivatives, centaureolides A (**14**) and B (**15**) isolated from *Centaurea pungens*. This herbaceous plant can be found on rocky and sandy pasturages in Algeria and Mauritania, occurring throughout the Sahara area and used in folk medicine for the treatment of respiratory infections. Isolated lactones are representatives of a group of sesquiterpenes containing l-proline residue. In order to determine the legitimacy of using *C. pungens* in traditional medicine, the antibacterial activity towards Gram-negative *P. aeruginosa* DSM 50071 and *Pseudomonas fragi* DSM 3456 and Gram-positive *Listeria innocua* DSM 20649 were tested. The minimal inhibitory concentrations were reported as 100, 400, and 400 µg/mL, respectively [33]. 

*Vernonia blumeoides* Hook f. is a perennial herb from the Asteraceae family growing across Northern Nigeria. The plant is used in Northern Nigerian ethnomedicine and is a rich source of biologically active substances. Its crude extract exhibited various treatments for diarrhea and malaria. One of the eudesmanolide sesquiterpene lactones—blumeoidolide-A (**16**), isolated from the aerial parts of this plant, demonstrated limited but interesting antibacterial activity against *B. subtilis* ATCC 6633 and two *Staphylococcus* strains [34] (Table 1). 

*Vernonanthura nudiflora* (Less.) H. Rob., is a species native to South America, occurring in Argentina, Brazil, and Uruguay. The crude extract and dichloromethane fractions together with isolated hirustinolides piptocarphins A and B (**17**, **18**) were subjected to antimicrobial evaluation. Interestingly, while the crude extract was inactive against almost all tested bacterial strains, the dichloromethane fraction was active towards *Mycobacterium tuberculosis*, *Enterococcus faecalis*, and *Aeromonas hydrophila*, with an MIC of 62.5, 125, and 125 µg/mL, respectively. Whereas the mixture of piptocarphins A and B (**17**, **18**) isolated from the dichloromethane fraction showed selective and promising activity against *M. tuberculosis* with an MIC of 15.6 µg/mL [35]. 

Gastrointestinal infections worldwide are the most common cause of diarrhea and are an important cause of morbidity and mortality in developing countries. Diarrheal diseases (DD) cause millions of deaths. There is a wide range of pathogens that can cause DD, including enterotoxin-producing bacterial strains such as *Escherichia coli*, *Salmonella typhi*, *Vibrio cholerae*, *Shigella sonnei*, and *Shigella flexneri*. Some *Decachaeta* species are used in traditional medicine in Mexico and Central American countries to treat gastrointestinal disorders such as diarrhea and abdominal pain [36,37,38]. Fractionation of the dichloromethane extract of *D. incompta* leaves using antiprotozoal and antimicrobial tests revealed the importance of incomptin A (**19**) and B (**20**). Incomptin A shows antiprotozoal activity against *Vibrio cholerae*. Incomptin B is an effective antibacterial sesquiterpene lactone against Gram-negative strains of chloramphenicol-resistant bacteria, including, *E. coli*, *S. sonnei*, and *S. flexneri* [37].

In addition to sesquiterpenes, diterpene compounds containing lactone moiety also have potent antimicrobial activities. They can be isolated from *Vitex vestitaa* (Lamiaceae), which is a shrub widely distributed throughout southeast Asia. The isolation of the bioactive compounds was preceded by extensive screening of the extracts derived from tropical plants towards 15 Gram-positive and 26 Gram-negative bacteria species. Subsequent bioassay-guided purification of the dichloromethane extract of the leaves of *Vitex vestita* led to the isolation of six new labdane-type diterpenoids. New vitexolide A (**21**) exhibited the most potent antibacterial activity against *B. cereus*, *S. hemolyticus*, and several *S. aureus* strains with minimal inhibitory concentration values ranging from 6 to 12 μM (2–4 mg/mL). The authors also stressed that the presence of a β-hydroxyalkyl-γ-hydroxybutenolide subunit contributed significantly to antibacterial activity [39]. Another member of the Lamiaceae family is *Salvia leriifolia* Benth. This perennial plant occurs solely in hot, southern regions of the Khorasan and Semnan provinces of Iran and in traditional medicine, is used for the treatment of tuberculosis and eczema. From the *n*-hexane extract of the aerial parts of *S. leriifolia*, both a new and a known labdane diterpenoid containing a γ-lactone ring were isolated. The compounds were tested for activity against Gram-negative *E. coli* and Gram-positive *S. aureus* together with four methicillin resistant *S. aureus* (MRSA) strains. Compound **22** showed an MIC of 213 µM against methicillin resistant *S. aureus* [40]. 

### 2.2. Lactones Isolated from Other Sources

Apart from plants, various strains of fungi can also be a valuable source of new antibacterials. *Aspergillus hiratsukae* SCSIO 5Bn1003 is a strain derived from unidentified coral collected in the South China Sea. Its crude extract exhibited various antibacterial activities in the pre-active screening, including *Bacillus subtilis*, *Bacillus thuringiensis*, *Staphylococcus aureus, Vibrio alginolyticus* XSBZ14, multiresistant *Pseudomonas aeruginosa*, and *Micrococcus lutea*. Three new compounds were isolated from it, including two with a lactone ring and several whose structures were described previously. The isolated γ-lactones—dimethylincisterol A2 (**23**) and butyrolactone I (**24**), showed significant antibacterial activities against *B. subtilis*, with MIC values of 10.26 ± 0.76 µM and 5.30 ± 0.29 µM, respectively (Table 2) [42]. Another marine-derived *Aspergillus* strain (*Aspergillus fumigatus* HNMF0047) was reported to be a source of γ- and δ-lactone derivatives of the already known helvolic acid, which is classified as a fusidane-type antibacterial. From the EtOAc extract of the fermentation broth, five new and two already described lactones were isolated. The antimicrobial potential of those compounds was tested against *S. aureus*, *E. coli*, *B. subtilis*, and *Streptococcus agalactiae*. Compound **25,** as well as helvolic acid, showed antibacterial activity against *S. agalactiae* with MIC values of 64 and 8 μg/mL, respectively (with tobramycin as the positive control, MIC 32 μg/mL) [43]. 

The next interesting species of fungi, which is a source of biologically active lactones, is mangrove-derived *Penicillium*. From *Penicillium* sp. TGM112 strain, three penicilactones was resourced through subsequent bioassay-guided fractionation and isolation. Those new compounds with a 6,7-dihydroxyocta-2,4-dien skeleton were tested towards six pathogenic bacteria, *E. coli* ATCC 25922, *S. aureus* ATCC 25923, *S. albus* ATCC 8799, *Micrococcus luteus* ATCC 10240, *Vibrio parahaemolyticus* ATCC 17802, and *V. alginolyticus* ATCC 17749. Only Penicilactone A (**26**) exhibited the relevant antibacterial properties against *S. aureus* with an MIC value of 6.25 µg/mL [44].

A new sesquiterpene lactone named eut-Guaiane sesquiterpene (**27**) was isolated from *Eutypella* sp. derived from the soil of the London Island of Kongsfjorden of Ny-Ålesund District of the Arctic. This compound showed strong antibacterial activities compared to the control ampicillin in the agar well diffusion assay against *E. coli*, *B. subtilis*, and *S. aureus* [45].

The higher fungi can also be an important source of antibacterial substances. In large-scale screening tests, the antibacterial activity of 160 extracts of 40 mushroom species, collected in Hungary, against standard bacterial strains and clinical isolates was checked [46]. Among these, the extracts of *Tapinella atrotomentosa* (Batsch) Šutara exhibited not only a broad-spectrum of antimicrobial activity on Gram-positive and Gram-negative pathogens but demonstrated also a significant inhibitory activity against resistant bacterial strains. The antibacterial lactones found in this chloroform extract are osmundalactone (**28**) and 5-hydroxy-hex-2-en-4-olide (**29**). These two fairly small molecules possess significant antibacterial activity against multiresistant *Acinetobacter baumannii* (MIC value of 10 and 6 µg/mL, respectively) and extended-spectrum β-lactamase (ESBL)-producing *E. coli* (MIC value of 10 µg/mL for both tested compounds) [47].

The vast majority of lactones showing antimicrobial properties are γ- and δ-lactones. However, there are also reports of ε-lactones showing antibacterial potential. Penicillilactone A (**30**) was isolated from the sponge-derived (*Haliclona* sp.) fungus *Penicillium* sp. LS54 as a first natural product containing a 7-membered lactone ring fused with a furan core. This interesting compound exhibited antibacterial activity (MIC value of 8 μg/mL) towards an aquatic pathogen *Vibrio harveyi* [48].

Abyssomicin C (**31**) from actinomycetes found in deep-sea sediment samples shows activity against Gram-positive bacteria, such as methicillin- and vancomycin-resistant strains of *S. aureus* (Table 2). Abyssomicin C acts by preventing the conversion of chorismate to *p*-aminobenzoic acid (*p*ABA), a precursor in the biosynthesis of tetrahydrofolate. This pathway appears to be of significant interest for the development of new antibiotics, as it is directly related to folic acid biosynthesis, which is established in plants, fungi, prokaryotes, and apicomplexa parasites (*Plasmodium, Toxoplasma*) but not in vertebrates [22].

β-Lactone rings occur infrequently in nature but are constituents of several different natural product classes. Structurally similar to β-lactam rings, they are effective electrophiles able to form covalent linkages with the nucleophilic residues of target proteins and possess significant therapeutic value as hydrolase inhibitors [49]. Obafluorin (**32**) is the structurally unique β-lactone, produced by *Pseudomonas fluorescens* ATCC 39502, active against both Gram-positive and -negative pathogens. Scott et al. proposed threonyl-tRNA synthetase as its biological target [50].

## 3. Synergistic Antimicrobial Activity

**Figure 2:**
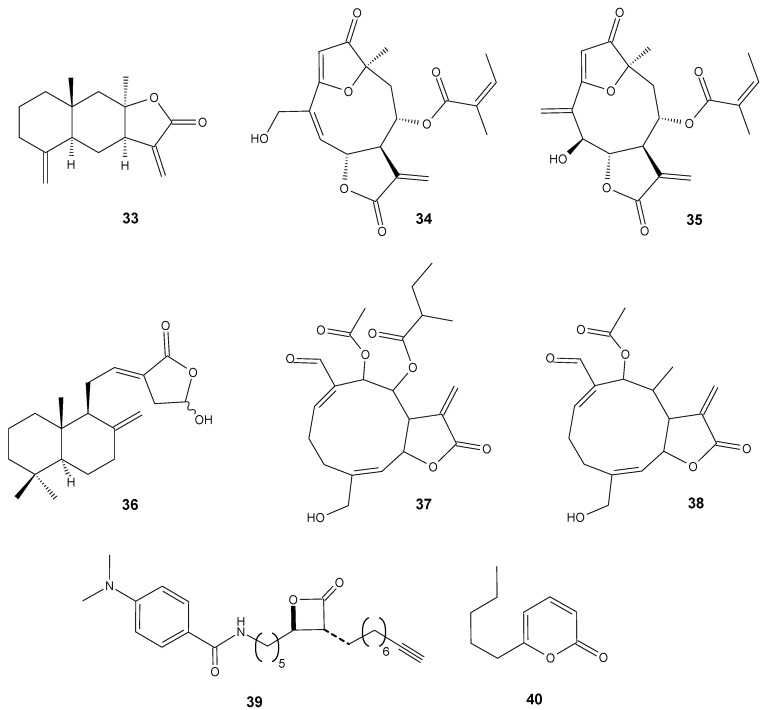
Lactones **33**–**40**.

Even though almost 100 years have passed since the discovery of penicillin by A. Fleming, β-lactam antibiotics are still the group of the most commonly used antibacterial drugs, both for home treatment and for those requiring hospitalization [51]. Unfortunately, the emergence of strains resistant to these antibiotics is often associated with the activity of β-lactamase or as with methicillin resistant *S. aureus*, the spreading of genes *mecA* encoding the penicillin-binding protein 2a (PBP2a). This enzyme is responsible for crosslinking the peptidoglycans in the bacterial cell wall. PBP2a has a low affinity for β-lactams and this directly leads to resistance to this class of antibiotics [52]. The therapies that combine β-lactam antibiotics and inhibitors of the β-lactamase activity are currently attracting great interest as an effective method for combating drug-resistant bacteria [21]. In this strategy, often antibiotics and antibiotic adjuvants are mixed to ensure their prolonged activity. The drawback of this approach is related to these inhibitors’ intrinsic antibacterial activity which may induce the development of further bacterial resistance. Interestingly, the combination of antibiotics and compounds that do not exhibit antimicrobial properties but can inhibit β-lactamase activity were also considered. There are several examples of combining β-lactam antibiotics with certain lactones to achieve a synergistic effect. One of them is the combination of penicillin G and isoalantolactone (IAL) (**33**) (Figure 2). The tests were performed on 21 β-lactamase-positive *S. aureus* strains which were obtained from porcine samples or purchased from American Type Culture Collection and included 15 methicillin resistant *S. aureus*. For all tested microorganisms with identified β-lactamase activity, the MIC values presented for penicillin G alone were significantly higher than those registered for the mixture of antibiotics and IAL. The authors presented their results as fractional inhibitory concentration—FIC index. Tt was calculated as (MIC value of IAL alone/MIC value of IAL in combination) + (MIC value of antibiotic alone/MIC value of antibiotic in combination). When the FIC index values are below 0.5, it is considered as a synergistic effect; between 0.5 and 1 is an additive; in range from 1 to 2, it represents no interaction; and above 2 is defined as antagonistic. For this study, all FIC indexes of penicillin G, in combination with IAL, were less than 0.5 and ranged from 0.10 to 0.38. Additionally, the effectiveness of the combined therapeutic effect was tested in vivo on a mouse model of intranasal lung infection. The results indicated that the survival rate of *S. aureus*-infected mice increased significantly from 35.29% to 88.24% within 144 h following multiple compound therapy approach [53].

Another example applies to sesquiterpene lactones isolated from *Centratherum punctatum* Cass. from the Asteraceae family. The two already described lactones isolated from the dichloromethane leaf extract identified as centratherin (**34**) (Figure 2) and 5-epi-isocentratherin (**35**) together with ampicillin were subjected to this study. The tests were performed towards two Gram-negative drug-resistant pathogens, β-lactamase (TEM-1)-producing *E. coli* ATCC 25218, and beta-lactamase (SHV-18)-producing *Klebsiella pneumoniae* ATCC 700603. The compounds tested individually showed low or no antibacterial activity. However, when ampicillin and centratherin (**34**) were combined, they showed synergistic interaction against both bacterial strains. The individual MIC values for centratherin and ampicillin were 5000 and 1250 μg/mL, respectively, for *E. coli*, and 5000 and 2500 μg/mL for *K. pneumoniae*. In combination, they reduced to 625 and 78 μg/mL for *E. coli*, and 1250 and 78 μg/mL for *K. pneumoniae*. Moreover, the FIC index indicated the synergistic effect of the compounds used in the experiment with values ranging from 0.185 for *E. coli* to 0.28 for *K. pneumoniae* [54].

Coronarin D (**36**) (Figure 2) has also been shown to have a synergistic potential with various antibiotics. This labdane-type diterpene consists of a 4-hydroxy-γ-lactone moiety combined with a decalin ring and exhibits antifungal and cytotoxic activity against cancer cells. In the present study, the activity of coronarin D and different antibiotics were tested against three strains of Gram-negative bacteria (*Pseudomonas aeruginosa*, *Escherichia coli*, and *Salmonella typhimunium*), four strains of Gram-positive bacteria (*S. aureus*, *S. epidermidis*, *E. faecalis*, and *B. cereus*), and several yeast and fungi strains. The results indicated that coronarin D was weakly active against the tested fungi, inactive against the tested Gram-negative bacteria, and active towards the Gram-positive bacteria. The authors adopted an approach in which they wanted to combine the positive antibacterial effect of coronarin D and nine commercially used antibiotics on Gram-positive bacteria. The results showed those synergistic or partial synergistic effects in most of the tested combinations. The lowest FIC index value was registered for the combination of coronarin D and gentamicin against *E. faecalis*—0.16. A strong synergistic effect was also observed for the mixture of coronarin D–gentamicin against *S. epidermidis* (0.19), *S. aureus*, and *E. faecalis* (0.25). For the coronarin D and oxacillin mixture, the FIC index reached 0.19 towards *S. epidermidis* and 0.25 against *B. cereus* and *S. aureus* [55].

Cartagena reported the synergistic interactions between some melampolide-type lactones and oxacillin or gentamicin against pathogenic strains of *S. aureus* and *E. faecalis.* The sesquiterpene lactones were isolated from *Acanthospermum hispidum* DC., a shrub native to northern Argentina. The plant is used in ethnomedicine against infections, and as a diuretic, abortive, and insect repellent, and is a rich source of biologically active substances. In previous research, the authors found that the sesquiterpene lactones isolated from *A. hispidum* exhibited selective antibacterial activity against Gram-positive human pathogenic strains of bacteria, being harmless on *Lactobacillus* [56,57]. Those findings led the authors to the idea of combining the antimicrobial potential of isolated lactones and two commonly used antibiotics. The most promising interactions were observed when acanthospermal B (**37**) (Figure 2) was combined with gentamicin on an ex vivo culture of methicillin-resistant *S. aureus*. The synergistic effect was manifested in a significant MIC reduction in acanthospermal B (312.5 to 78.1 μg/mL) and gentamicin (120 μg/mL to 3 μg/mL), and the value of the FIC index reached 0.3. Compound (**38**) (Figure 2) improved the antibiotic potency of oxacillin towards ampicillin-resistant *E. faecalis*. For this pair of tested compounds, the FIC index value reached 0.1 [58].

According to the World Health Organization, tuberculosis currently remains the second leading cause of death due to a single infectious agent—*Mycobacterium tuberculosis* [59]. The spread of antibiotic resistance is a major challenge for the treatment of *M. tuberculosis* infections. In addition, drug efficacy is often restricted by the limited permeability of the cell membrane. First-line antibiotics inhibit mycomembrane biosynthesis, leading to rapid cell death. A β-lactone named EZ120 (**39**) (Figure 2) as a mycolic acid mimetic inhibits serine hydrolases involved in their biosynthesis and, as a result, exhibits potent antibacterial and bactericidal activity. Simultaneous administration with first-line antibiotics increases their potency against *M. tuberculosis* by more than 100-fold, thus demonstrating therapeutic potential [60].

The fungal metabolite 6-pentyl-α-pyrone lactone (**40**) (Figure 2) is produced by the *Trichoderma* species and shows a strong antifungal activity against selected *Fusarium*, *Penicillium*, and *Aspergillus* strains [61]. The antimicrobial properties of this compound are much lower; however, in combination with zinc oxide nanoparticles, (ZnONPs) they exhibited a synergistic effect against multidrug-resistant *Enterobacterales* recovered from urinary tract infections in humans. ZnONPs arouse great interest in the field of biomedicine, especially as anticancer and antibacterial compounds. The antimicrobial effect of ZnONPs against the investigated isolates is shown in the MIC values ranging from 0.015 to 32 µg/mL. Nevertheless, the MICs decreased 5–12-fold for 6-pentyl-α-pyrone lactone (**40**) and 3–11-fold for ZnONPs, after their combination [62].

## 4. Quorum Sensing

**Figure 3:**
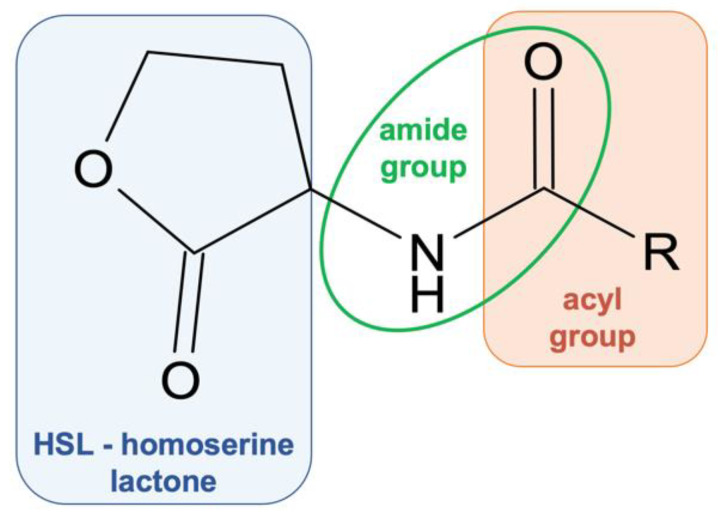
General structure of *N*-acyl-homoserine lactones.

Quorum sensing (QS) is a specific cell-to-cell signaling used by bacteria for communication and allows it to function as a population by regulating gene expression. QS controls different aspects of pathogenicity such as susceptibility to antibiotics, biofilm formation, and cell adhesion. Such a wide range of activities regulated by QS provides an interesting target for the design of new types of substances modulating bacterial “behavior”. Both Gram-positive and Gram-negative bacteria are regulated by QS signaling. However, in the case of Gram-negative bacteria, *N*-acyl-homoserine lactones (AHSLs) (Figure 3) are the main players [63,64]. 

**Figure 4:**
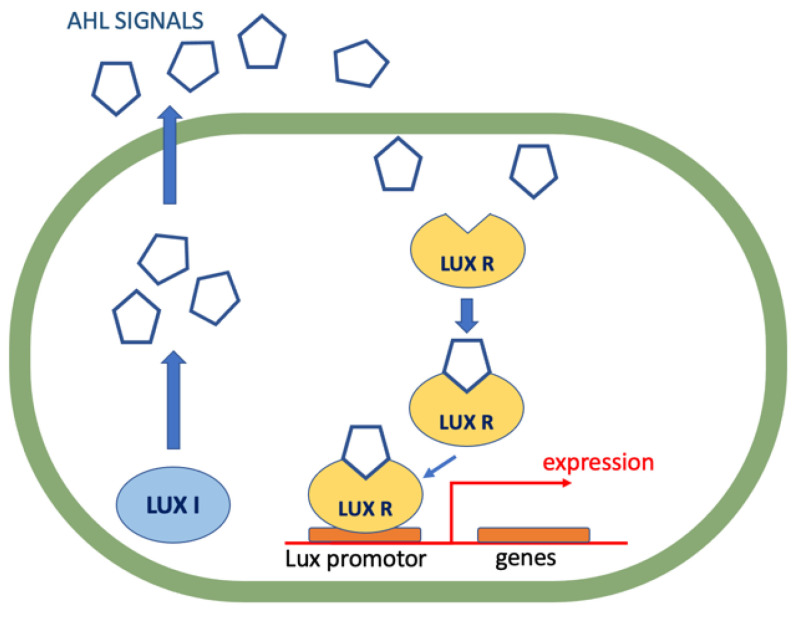
The LuxI/LuxR quorum sensing system of Gram-negative bacteria.

Pseudomonas aeruginosa is one of the pathogens that, under the acronym ESKAPE (*Enterococcus faecium, Staphylococcus aureus, Klebsiella pneumoniae, Acinetobacter baumannii, Pseudomonas aeruginosa* and *Enterobacter* species), is on the WHO’s list of bacteria that pose the greatest threat with an urgent need for developing new treatments [65,66]. It is an opportunistic pathogen that can cause multidrug-resistant infections. Nosocomial infections with high morbidity and mortality, mainly among immunocompromised patients and intensive care units, are a particular problem. As with many Gram-negative bacteria, also for *P. aeruginosa*, LuxI/R signaling systems are involved in QS [67,68] (Figure 4). 

**Figure 5:**
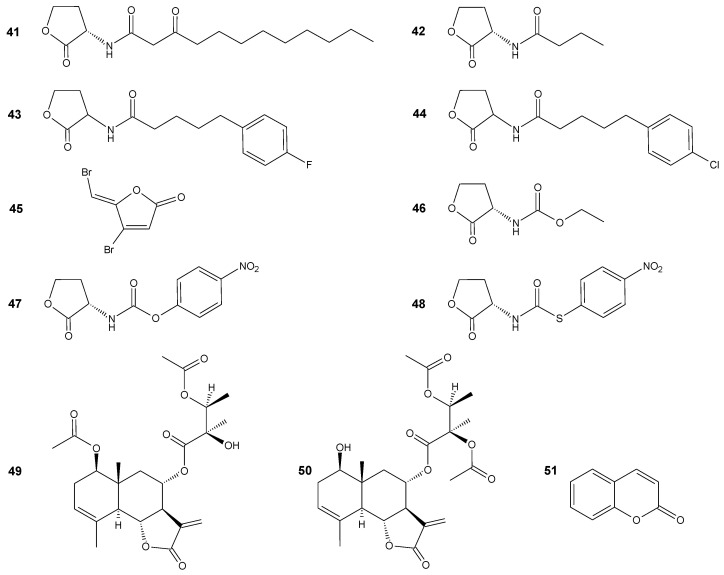
Lactones **41**–**51**.

LuxI-type enzymes produce acyl homoserine lactones (AHSL) which are small signaling molecules. LuxR-type proteins act as AHSL receptors and serve as transcriptional regulators. AHSLs consist of a homoserine ring attached with an acyl side chain which may have an oxo- or hydroxyl group at the C-3 position. In general, AHSLs differ mainly by the length and substitution of this acyl chain. For *P. aeruginosa*, two systems (LasI/R and RhlI/R) function within the LuxI/R signaling cascade and two AHSLs are involved as signaling molecules: *N*-(3-oxododecanoyl)homoserine lactone (**41**) and *N*-butyrylhomoserine lactone (**42**) (Figure 5) [69]. One interesting aspect of the design of QS modulators is the modification of the HSL side chain to obtain anti-QS compounds. Kalaiarasan et al. reported the synthesis of two lactones which act as biofilm formation inhibitors. The structure of *N*-(4-{4-fluoroanilno}butanoyl)-l-homoserine lactone (**43**) and *N*-(4-{4-chlororoanilno}butanoyl)-l-homoserine lactone (**44**) (Figure 5) was designed on the basis of in silico docking analysis with LasR protein. Those compounds were designed to exhibit a higher hydrogen bonding potential to the LasR receptor. Inhibition of biofilm formation was performed on extensively drug-resistant *P. aeruginosa*. The experiments proved that both compounds have the potential to serve as biofilm inhibitors via disabling the QS system [70].

A different approach can be related to modulations of homoserine lactone synthases activity (LasI and RhlI). To answer the question of whether modifications of the structure of HSL derivatives may affect the activity of these enzymes, more than 90 compounds with different structural features were taken into consideration. For this extensive study, Shin et al. analyzed several groups of HSL analogs such as d- and l-homoserine lactones, d- and l-homoserine tiolactones, sulfonamides, and compounds without lactone moiety. This led to the conclusions that for both lactones and thiolactones, the d-configuration of the stereogenic center at the head group increased the inhibition potency towards the RhlI synthase. The strong inhibition activity was also noticed for longer, unsubstituted acyl-chain thiolactones. In contrast, the analogs with thiolactone head group attached to short and medium 3-oxoacyl-chains are enzyme activators [71]. 

Some studies also combine the traditional antibiotics with antivirulence factors to benefit from virulence suppression and effective pathogen removal. Furanone C-30 (**45**) (Figure 5) is a synthetic, dibrominated AHSL analog which acts as a bacterial quorum sensing inhibitor by competing with the AHSL for binding to the LasR receptor in a concentration-dependent manner. In the study, the effectiveness of combined treatment with Furanone C-30 (**45**) and four clinically relevant antibiotics such as ciprofloxacin, colistin, meropenem, and tobramycin was evaluated. The model microorganism used, in this case, was antibiotic-resistant *P. aeruginosa*. Antagonistic interactions were observed mainly for furanone–meropenem and furanone–ciprofloxacin combinations. Whereas for furanone–colistin and furanone–tobramycin, significant synergistic effect was observed [72]. 

Additionally, for *Vibrio fischeri*, AHSLs act as a QS regulator by binding to their cognate transcriptional regulators belonging to the LuxR family. The AHSL structural analogs which belong to the carbamate, thiocarbamate, and hydrazide family were synthesized and evaluated as QS modulators. Ethyl-substituted carbamate (**46**) (Figure 5) exhibited a weak agonistic activity on the *V. fischeri* QS system, whereas compounds with longer side chain or with benzyl substituents exhibited significant antagonistic activity. The compounds with the highest activity were 4-nitrobenzyl carbamate (**47**) and thiocarbamate (**48**) (Figure 5) which displayed an IC_50_ value of about 20 µM. Docking experiments carried out on the LuxR model indicated the additional hydrogen bond formation between the carbamate group and Tyr70. The most active compound with a *p*-nitro benzyl substituent (**47**) exhibited additional interactions with Lys178 [73]. 

Not only the HSL analogs may be targeted as virulence inhibitors. Sesquiterpene lactones may also modulate QS. Some eudesmanolide-type sesquiterpene lactones that were isolated from *Vernonia blumeoides* were tested as quorum sensing inhibitors by violacein (purple pigment) inhibition assays with *Chromobacterium violaceum* and *Agrobacterium tumefaciens* biosensor systems and in silico molecular docking. The first test showed ≥ 80% inhibition of violacein production by blumeoidolide A (**49**) (Figure 5) in concentrations ≥ 3.6 mg/mL and 0.071 mg/mL for blumeoidolide B (**50**) (Figure 5). Agar diffusion double ring assays indicated that blumeoidolides A and B modulated the CviI (LuxI synthase homologue) activity and docking experiments suggested that both lactones have a tendency to inhibit CviR and CviR’ (LuxR homologues) with varying binding affinities [74].

Coumarin (**51**) (Figure 5) also has potential to control the virulence behavior of a broad spectrum of bacterial pathogens. It inhibited biofilm formation in 1.36 mM concentration for *P. aeruginosa* PA14, *E. coli* MUH, *V. anguillarum, E. tarda*, and *S. aureus* NCDO949 strains. The fact that coumarin has the ability to inhibit biofilm formation in both Gram-negative and Gram-positive bacteria could support its role as a universal QS inhibitor. The effect of coumarin towards the biosensor system was evaluated. The microorganisms (*Serratia marcescens*, *C. violaceum*, and *A. tumefaciens*) were selected to target the short, medium and long chain AHSL-mediated processes. Coumarin exhibited a strong QS inhibition activity against all three biosensors and additionally inhibited QS system-associated pigment production on all three biosensor strains at 100 and 125 μg [75].

## 5. Design and Synthesis

Currently, there are two main approaches to designing biologically active compounds. One is to modify known structures with proven activity. The other is to use computer techniques to design new compounds active towards molecular targets.

**Figure 6:**
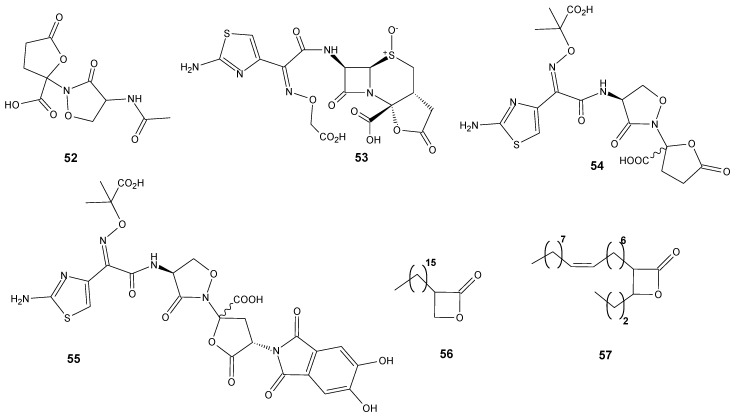
Lactones **52**–**57**.

Traditional chemical synthesis is one of the most frequently used methods to modify the structure of biologically active compounds. The purpose of these transformations is to obtain pharmaceuticals with greater efficiency or for which microorganisms have not yet developed resistance mechanisms. A great example is the tricyclic β-lactams in which the additional ring is specifically a lactone ring. They were designed as analogs of already known Lactivicin (**52**) (Figure 6), the only clinically relevant, naturally occurring, penicillin-binding protein inhibitor [76]. The designed compounds were evaluated for in vitro antibacterial activities against carbapenem-resistant *Enterobacterales*. The most active compound **53** (Figure 6) showed significant antibacterial potential against class C β-lactamase producers while maintaining antibacterial activities against class A, B, and D β-lactamase producers. Evaluation of antibacterial activities against clinical isolates of *E. coli*, *Enterobacter cloacae*, *K. pneumoniae*, *Enterobacter aerogenes*, or *Citrobacter freundii* showed that **53** exhibited lower MIC_90_ values than those of the already approved combinations of β-lactam and β-lactamase inhibitor and showed a potent therapeutic efficacy in the neutropenic mouse lung infection model [77]. The modification of Lactivicin with a 2-aminothiazol side chain (**54**) and, additionally, with catechol-type siderophore (**55**) (Figure 6) increased its potency 1000-fold against *Stenotrophomonas maltophilia*, a species notoriously resistant to antimicrobial drugs. Calvopiña et al. reported that the MIC_90_ values for **55** against a wide range of extensively drug-resistant clinical *S. maltophilia* isolates were about 0.063 µg/mL. Lactone **55** was not observed to have an inhibitory effect on the production of the L1 and L2 lactamases. Moreover, no reduced hydrolysis of lactone **55** by L1 and L2 was observed. Therefore, the authors believed that such high activity of the obtained derivative was related to substantially increased penetration via siderophore uptake [78].

On the basis of described by Lehmann et al. [60] of the activity of β-lactones towards *M. tuberculosis*, new modification of the structure was also designed and some α,β-disubstituted β-lactones were tested towards different mycobacterial pathogens. Among the 16 β-lactones, 3-hexadecyloxetan-2-one (**56**) (Figure 6) exhibited significant activity against *M.* *abscessus*, whereas most of the β-lactones showed promising activities against *M. marinum*, similar to that of the isoniazid. There were 6 compounds found to be active against *M. tuberculosis*, with *trans*-(*Z*)-3-(hexadec-7-en-1-yl)-4-propyloxetan-2-one (**57**) (Figure 6) being the best growth inhibitor with an MIC_50_ value reaching 19.7 µg/mL [79].

**Figure 7:**
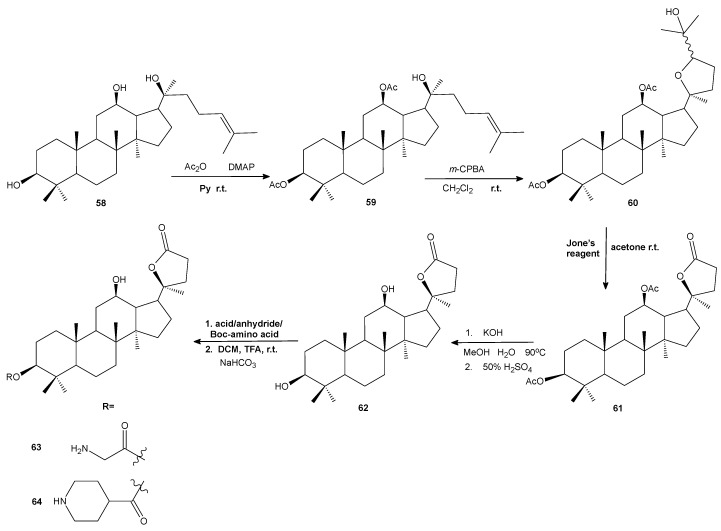
The synthesis of lactones **63** and **64**.

Based on the previous findings on Ocotillol antibacterial activity (triterpene isolated from *Fouquieria splendens* Engelm.), a series of novel ocotillol-type lactone derivatives were designed and synthesized. The five-step synthesis started from 20(*S*)-protopanaxadiol (PPD, **58**) and included the protection of hydroxyls by acetic anhydride, epoxidation followed by nucleophilic addition in situ to target the tetrahydrofuran derivative, oxidation by Jones reagent to provide acetylated lactone, and, afterwards, removing the protecting groups and subsequent reaction with acids, anhydrides or amino acid to afford the target Ocotillol derivatives (Figure 7). Among the tested derivatives, compounds **63** and **64** were found to be the most active with minimum inhibitory concentrations of 1–4 μg/mL against Gram-positive bacteria, and at the same time, they exhibited low cytotoxicity against normal and cancer cell lines. The synergistic effect was tested with kanamycin and chloramphenicol and results showed that compounds **63** and **64** enhance the susceptibility of MRSA USA300 and *B. subtilis* 168 (FICI < 0.5). The authors suggested that the most active lactone **64** may exert its antibacterial effect by damaging bacterial cell membranes and disrupting the function of DNA [80].

**Figure 8:**
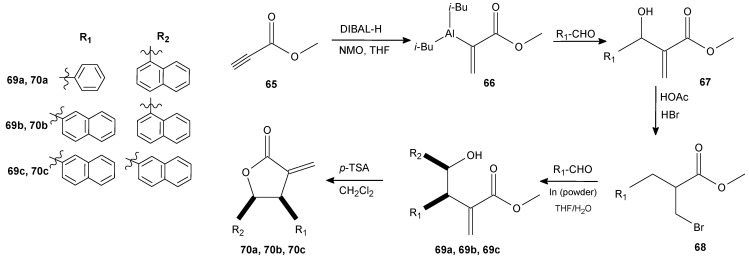
The synthesis of lactones **70a**–**c**.

The structure and the activity of naturally occurring α-methylene-γ-lactones was the starting point for the design of an α-methylene-γ-butyrolactones series bearing aryl moieties at the β- and γ-positions. The compounds were synthesized via allylboration or allylindation reactions and subsequently tested towards MRSA. The activity of the most potent **70a**, **70b**, and **70c** (Figure 8) were comparable to vancomycin and linezolid, the drugs of last resort for MRSA infections. The mammalian cells (Caco-2 and HaCaT) showed acceptable tolerability against the most promising compounds. Additionally, butyrolactones also exhibited rapid bactericidal killing kinetics against MRSA, outperforming the vancomycin. The lactones were also tested for inhibiting staphylococcal protease production and turned out to be more active than vancomycin [81]. 

**Figure 9:**
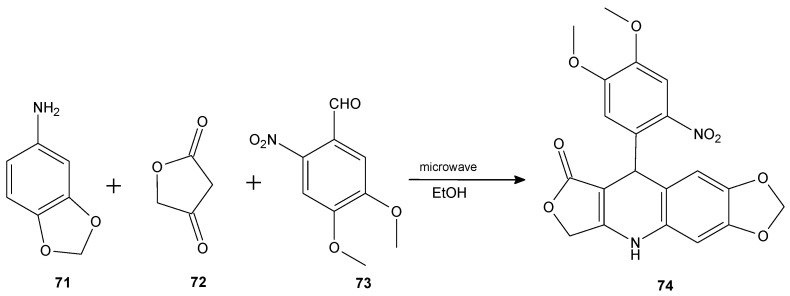
The synthesis of lactone **74**.

The combination of 1,4-dihydroquinoline and lactone rings results in 7-azo synthetic analogs of lignan lactones. This group of compounds is well-known for their biological potential but still the antibacterial properties are not thoroughly researched. Therefore, a series of 39 lactone 1,4-dihydroquinoline derivatives was obtained via a microwave-assisted reaction between aniline derivative **71**, tetronic acid **72**, and aromatic aldehydes (e.g., **73**) performed in ethanol, and their antibacterial activity was evaluated against several bacterial strains including *M. tuberculosis*, *M. avium*, and *Mycobacterium kansasii*. Overall, compound **74** (Figure 9) was the most active against the three strains of *Mycobacterium* with MIC values ≥125 µg/mL. Based on structure–activity relationship studies, the authors noticed that the presence of a nitro group on the benzylic ring and a methylenedioxy group on the dihydroquinoline ring enhanced the antibacterial activity of the derivatives [82].

**Figure 10:**
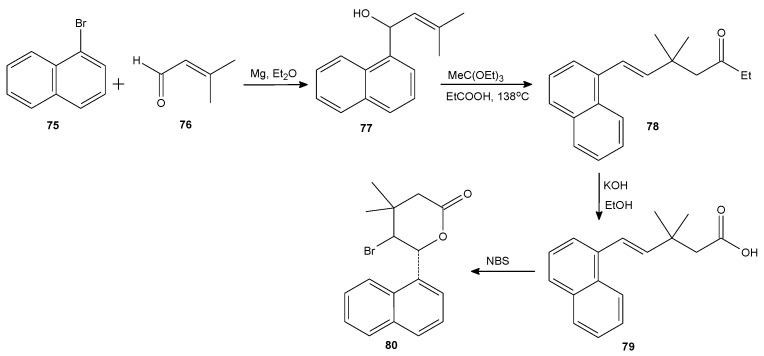
The synthesis of lactone **80**.

Some other aromatic lactones were obtained by Kamizela et al. in a four-step chemical synthesis (Figure 10). Starting with the Grignard reaction between 3-methyl crotonaldehyde and three aromatic magnesium bromides (α-naphthylene, phenyl, and p-fluorophenyl), the three corresponding unsaturated secondary alcohols were obtained and reacted via the Johnson–Claisen rearrangement to unsaturated esters. After the following hydrolysis, the acids were subjected to three halolactonization reactions resulting in iodo-, bromo-, and chloro-δ-lactones. Their antibacterial activity was evaluated against *E. coli* ATCC 8739 and *S. aureus* ATCC 65389. The most active lactone **80** in concentration 50 µg/mL decreased the average number of colony-forming units (CFUs), by about 50% for both microorganisms, compared to the number of CFUs in the control [83]. A similar synthetic pathway was applied by Kowalczyk et al. to achieve a series of aromatic δ-lactones. The starting Grignard reaction was performed this time with crotonaldehyde. An in-depth study was also carried out on the antibacterial activity of those compounds against *E. coli* strains diversified in terms of occurrence and length of lipopolysaccharides (LPS) in its structure. The most sensitive was the *E. coli* strain with the longest LPS. Moreover, oxidative damage of bacterial DNA was also analyzed and the damage values were comparable to those obtained for ciprofloxacin, bleomycin, and cloxacillin [84].

**Figure 11:**
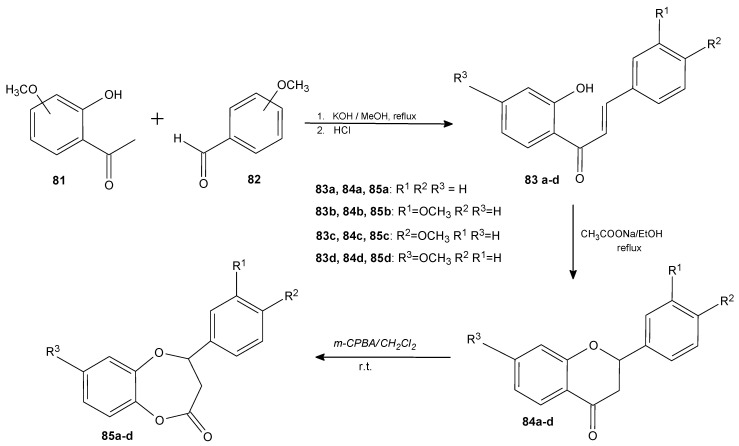
The synthesis of lactone **85a**–**d**.

Gładkowski et al. presented the synthesis of γ-oxa-ε-lactones in which chalkones and flavanones were the intermediates. (Figure 11). Starting with Claisen–Schmidt condensation under alkaline conditions between 2′-hydroxyacetophenones (**81**) and benzaldehydes (**82**), the cyclisation of the obtained 2′-hydroxychalcones (**83a**–**d**) was performed in the presence of sodium acetate. In the third step, the synthesized flavanones (**84a**–**d**) were oxidized with *m*-CPBA to gain results corresponding ε-lactones (**85a**–**d**). All the compounds were tested for their antimicrobial activity towards a wide range of pathogenic bacteria (*E. coli*, *B. subtilis*, *S. aureus*), fungi (*Fusarium graminearum*, *Aspergillus niger*, *Alternaria* sp.), and yeast (*Candida albicans*). Lactone **85a** exhibited the broadest spectrum of activity especially towards filamentous fungi and yeast. The methoxy substitution in the 3′ (**85b**) and 4′ (**85c**) position of the B ring enhanced the antibacterial activity, whereas substitution in the 7 position of the A ring (**85d**) enhanced antifungal properties. In most cases, the introduction of lactone function increased the activity of the lactones compared to its chalcone and flavanone precursors [85]. 

## 6. Biotransformations as a Method for Obtaining New Lactone Derivatives

**Figure 12:**
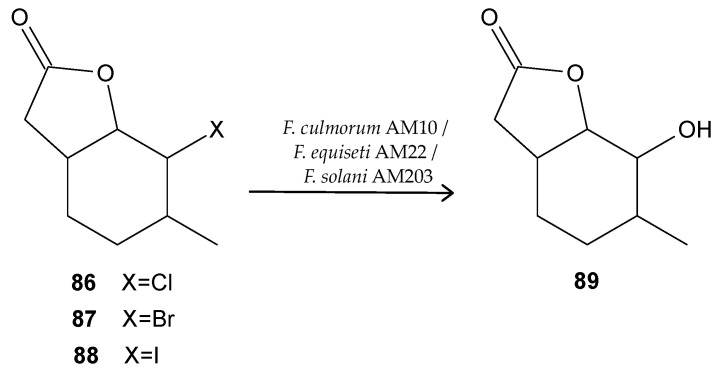
Lactones **86**–**89**.

Microbial transformations of chemical compounds are an interesting tool for obtaining new derivatives. Currently, purified enzymes are very often used for this purpose, but the use of whole cells of microorganizms is an interesting alternative. Crucial advantages are no need to isolate the biocatalyst and no need to add expensive cofactors. It is possible to obtain reaction products more easily in cascade processes. Biotransformations can also be very useful for obtaining derivatives with interesting biological activities. Some bicyclic γ-lactones with a methyl substituted cyclohexane ring (**86**, **87**, **88**) also exhibit antimicrobial potential. Those compounds were prepared by different halolactonization strategies. Iodolactonization of γ,δ-unsaturated acids was performed in the I_2_/KI mixture in aqueous NaHCO_3_. Bromo- and chlorolactonization was mediated by NBS and NCS, respectively. Interestingly, the obtained lactone compounds were subjected to the biotransformation reaction in which the enzymatic potential of selected filamentous fungi (*Fusarium culmorum* AM10, *Fusarium equiseti* AM22, *Fusarium solani* AM203) was used to modify the lactone structure. As a result of these experiments, the halogen atom was replaced by hydroxyl (**89**), regardless of the type of halolactone used as the substrate. The antimicrobial activity experiments indicated that these compounds have the ability to inhibit or limit the growth of bacteria, yeast, and fungi. However, the yeast and fungi were more sensitive and hydroxylactone was the least active compound. The most significant inhibitory effect on bacterial growth was shown by the halolactones with a methyl group at the C-3 position (compounds **86**, **87**, **88**) (Figure 12) [86].

**Figure 13:**
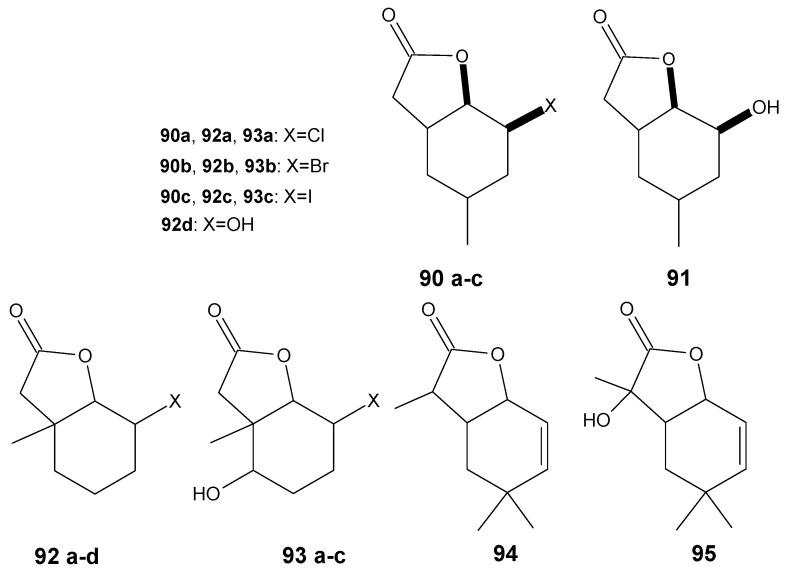
Lactones **90a**–**95**.

Some bicyclic lactones with cyclohexane moiety are well-documented antifeedants, especially towards storage product pests [87,88]. There are some examples for which this unique activity is combined with antimicrobial potential, such as for lactones with 4-methylcyclohexane system. Three bicyclic halolactones with a 4-methylcyclohexane ring were subjected to microbial transformation in order to obtain the hydroxyderivative in the hydrolytic dehalogenation process. All lactones, both substrate and biotransformation products, were able to inhibit the growth of the *S. aureus* strain. Complete inhibition was observed for lactones **90a**–**c**, and lactone **91** (Figure 13) showed a slight inhibition on the growth of this strain together with a significant extension of the adaptive phase (20–36 h) [89].

In other work, Wińska et al. presented similarly designed research in the subjects of synthesis and the transformations of halolactones with 6-methylcyclohexane (**92a**–**c**). Moreover, for these compounds, the biotransformation processes in the culture of *Fusarium equiseti* AM22 and *Yarrowia lipolytica* AM71 led to the hydrolytic dehalogenation product (**92d**), but also in *Penicillium vermiculatum* AM30 culture, the hydroxyl substituent was introduced at C-5 without removal of the halogen atom (**93a**–**c**). As in the studies discussed above, the same tendency was also noticeable in the tests of antibacterial activity. The halolactones were much more potent than hydroxylactone obtained as biotransformation product. The presented results may suggest that the presence of the halogen atom significantly increases the antimicrobial activity of this group of compounds [90]. Interestingly, for unsaturated lactone **94** and its biotransformation product, hydroxyderivative **95** (Figure 13), the tendency for antifungal activity was rather reversed. The most active was hydroxylactone **95** which, at 0.025 mg/mL, completely inhibited the growth of the *Fusarium linii* A3 strain [91].

## 7. Nafithromycin—New Promising Antibiotic

**Figure 14:**
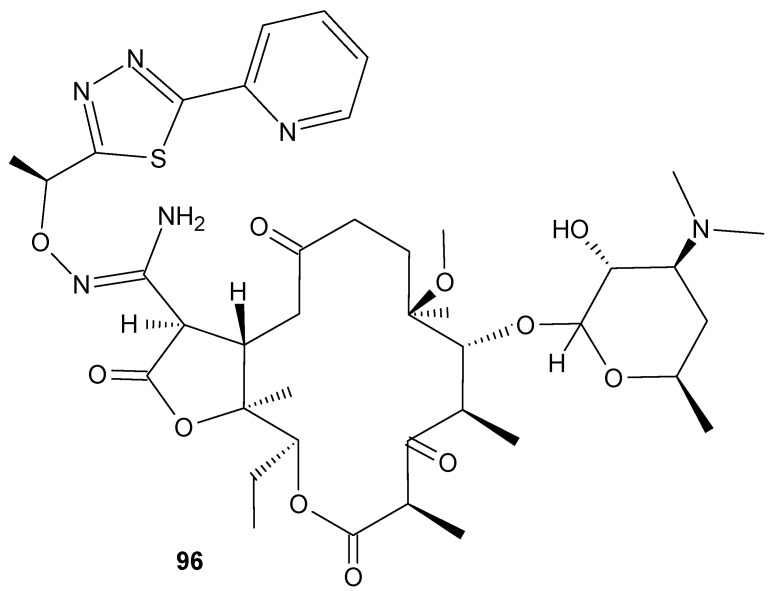
Nafithromycin (WCK 4873) (**96**).

Macrolide-resistant *S. pneumoniae* coharboring penicillin-resistant isolates are a current concern in community-acquired pneumonia in adults. Nafithromycin (WCK 4873) (**96**) (Figure 14) is a product from Wockhardt’s drug discovery program. It is a novel antimicrobial agent of the lactone ketolide class, under clinical development as an orally administered antibiotic for the treatment of community-acquired bacterial pneumonia and other respiratory tract infections. Nafithromycin is classified as a ketolide but has an additional γ-lactone ring instead of a carbamate moiety. That unique structural feature plays a key role in inhibiting RNA-dependent protein biosynthesis and enables nafithromycin to demonstrate potent activity against macrolide-resistant *S. pneumoniae* [92]. Nafithromycin also showed excellent activity against methicillin-susceptible *S. aureus* ATCC 29213, *E. faecalis* ATCC 29212, *S. pyogenes* and moderate activity against *H. influenzae*, and potent activity against *M. catarrhalis* [93,94,95]. Safety, tolerability, and pharmacokinetics during oral administration of nafithromycin was also tested in randomized, double-blind, placebo-controlled studies (registered at ClinicalTrials.gov at the no. NCT03926962 and NCT03979859). At first, single ascending oral doses of nafithromycin (100 to 1200 mg) were administered to human subjects under fasted or fed conditions to evaluated the effects of food on the bioavailability of nafithromycin. Single oral doses of nafithromycin were well-tolerated at all dose levels tested. There were no deaths or treatment-emergent serious adverse events reported and no withdrawals from the study due to an adverse event. No difference in tolerability was detected between the fed and fasted conditions. In the second study, multiple ascending oral doses of 600, 800, or 1000 mg of nafithromycin were administered once daily for 7 days under fed conditions. In this experiment, nafithromycin was also well-tolerated at all doses tested and no serious or severe adverse events were observed. In single-dose pharmacokinetic experiments, the highest geometric mean plasma concentrations were observed between 1 and 6 h postdose, and the mean maximum plasma concentration ranged from 0.099 to 1.742 mg/L. The experiments also led to the finding that plasma exposure to nafithromycin appeared to increase more than dose proportionally and appeared to be increased by the administration of food. In the multiple-dose study, the day 7 nafithromycin maximum plasma concentration ranged from 1.340 to 2.987 mg/L and the area under the concentration–time curve over the final dosing interval (AUC_0–24_) ranged from 13.48 to 43.46 h·mg/L. The steady state was achieved after 3 days in the group where doses of 600 and 800 mg was administered and after 4 days when 1000 mg was administered. On day 7 of dosing, the nafithromycin showed moderate accumulation. All data collected during this study support further development of nafithromycin [96].

## 8. Summary

The development of bacterial resistance to antibiotics and, consequently, the lack of effective antibiotic therapy is one of the greatest problems of medicine today. Some of the most serious related risks are longer hospital stays, higher medical costs, and increased mortality. Although antibiotic resistance occurs naturally, the overuse and misuse of antibiotics in human and animal therapy lead to the aggravation of the problem. Microbial resistance is increasing to dangerously high levels worldwide as new resistance mechanisms emerge and spread. The consequence of these processes is an urgent need for the continuous design and development of new antimicrobial agents. Given the total number of drugs approved each year on the global market, the number of new substances with antimicrobial activity is insufficient. Therefore, special attention should be given to research focusing on obtaining new antimicrobial substances that, in the long term, can be in the front line of the fight against the most dangerous pathogens. The solution to this problem will probably not be an antibiotic holy grail that is ideal in all cases. However, a much more restrictive use of antibiotics, development of combination therapies together with increased public awareness of the topic of resistance, and increased research funding linked to the discovery of novel antibacterials can certainly play a key role. Small size ring lactones are a very diverse group of compounds that can act by different mechanisms. In some cases, their mode of action is known, and further modification of their structure improves pharmacokinetic properties or broadens the spectrum of action. An example is nafithromycin, an antibiotic in phase III clinical trials. An interesting alternative is to search for new compounds active against other molecular targets, as in the case of exploiting the quorum sensing phenomenon and developing the possibility of using compounds with a lactone ring as its inhibitors. Inspirations for this research can be taken from nature, or it can come from molecular modeling and precise drug design, but the most important thing in this field right now is to find new therapeutics that are effective particularly against drug-resistant microorganisms. It is possible that among those future pharmaceuticals compounds with the lactone moiety will have their share.

## Figures and Tables

**Table 1:** Antimicrobial activity of lactones isolated from plants.

No.	Chemical Structure	Tested Microorganism	MIC	Source	Ref.
**3**	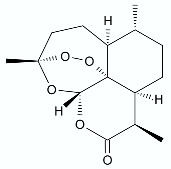 Artemisinin	*A. actinomycetemcomitans* *F. nucleatum* subsp. *animalis* *F. nucleatum* subsp. *polymorphum* *P. intermedia* *B. subtilis* *S. aureus* *Salmonella* sp.	14 mg/mL 14 mg/mL 14 mg/mL 14 mg/mL 14 mg/mL 14 mg/mL 14 mg/mL	*Artemisia annua* L.	[27,28]
**6**	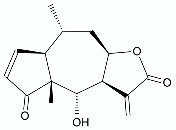 Helenalin	*S. aureus* ATCC 29740	NI	*Arnica montana*	[29]
**7**	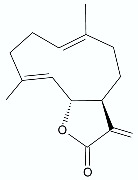 Costunolide	*M. tuberculosis* *M. avium* *H. pylori* *P. aeruginosa* IBRS P001	12.5 mg/L 128 μg/mL 100–200 μg/mL 0.5 mg/mL	*Saussurea lappa*	[13,26]
**8**	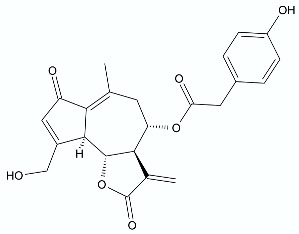 Lactucopicrin	*S. aureus* ATCC 11632 *P. aeruginosa* ATCC 27853 *P. aeruginosa* IBRS P001	0.16 mg/mL 0.31 mg/mL 0.50 mg/mL	*Lactuca virosa*	[26]
**9**	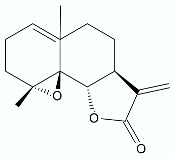 Partenolide	*S. aureus* (oral isolate) *S. aureus* ATCC 11632 *P. aeruginosa* ATCC 27853	0.08 mg/mL 0.16 mg/mL 0.31 mg/mL	*Tanacetum parthenium*	[26]
**10**	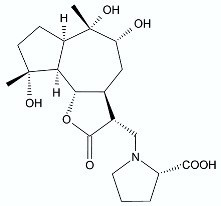	*S. aureus* *E. fergusonii*	0.32 μg /mL 1.7 μg /mL	*Anvillea garcinii*	[31]
**11**	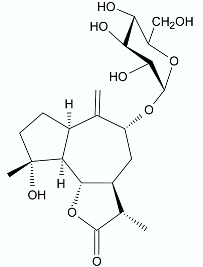	*S. aureus* *E. fergusonii*	1.4 μg/mL 3.5 μg/mL	*Anvillea garcinii*	[31]
**12**	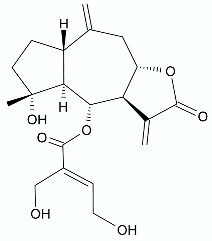	*P. aeruginosa* *S. aureus* *E. coli*	46.88 μg/mL 62.5 μg/mL 125 μg/mL	*Schkuhria pinnata*	[32]
**13**	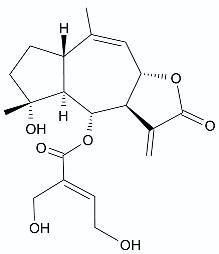 mixture				
**14**	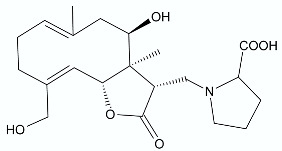	*P. aeruginosa* *P. fragi* *L. innocua*	100 μg/mL 400 μg/mL 400 μg/mL	*Centaurea pungens*	[33]
**15**	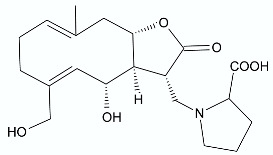	*P. aeruginosa*	100 μg/mL	*Centaurea pungens*	[33]
**16**	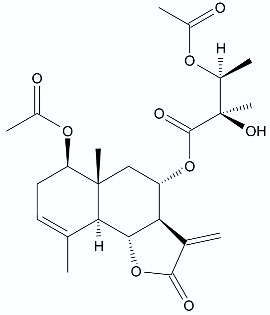 Blumeoidolide A	*S. aureus* ATCC 29213 *S. aureus* ATCC 43300 *B. subtilis* ATCC 6633	2.15 mM 4.30 mM 2.15 mM	*Vernonia blumeoides*	[34]
**17**, **18**	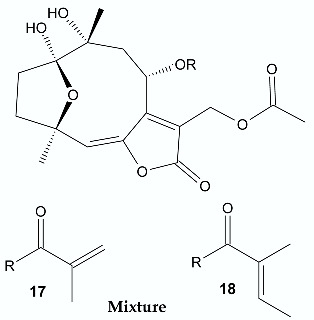 Piptocarphin A, Piptocarphin B	*M. tuberculosis* *E. faecalis* *A. hydrophila*	15.6 μg/mL 125 μg/mL 125 μg/mL	*Vernonanthura nudiflora*	[35]
**19**	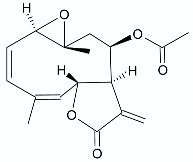 Incoptine A	*V. cholerae*	0.15 mg/mL	*Decachaeta incompta*	[37]
**20**	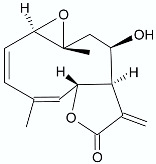 Incoptine B	*V. cholerae* *E. coli* *S. sonnei* *S. flexneri*	0.05 mg/mL 0.4 mg/mL 0.6 mg/mL 0.5 mg/mL	*Decachaeta incompta*	[37,41]
**21**	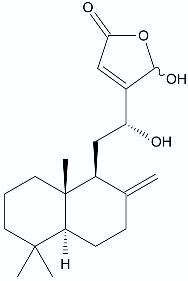	*B. cereus* CIP 6624 *B. cereus* N190 *B. cereus* N258 *B. cereus* N349 *B. subtilis* ATCC 66.33 *S. aureus* CIP 4.83 *S. aureus* CIP 53156 *S. aureus* CRBIP 21.21 *S. hemolyticus* CIP 81.56	12 mg/mL 6 mg/mL 6 mg/mL 6 mg/mL 6 mg/mL 12 mg/mL 24 mg/mL 12 mg/mL 6 mg/mL	*Vitex vestita*	[39]
**22**	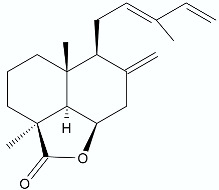	*E. coli* ATCC 25922 *S. aureus* ATCC25923 *MRSA strains* (clinically isolated)	314 μM 314 μM 213 μM	*Salvia leriifolia*	[40]

NI—MIC values not indicated.

**Table 2:** Antimicrobial activity of lactones isolated from other sources.

No.	Chemical Structure	Tested Microorganism	MIC	Source	Ref.
**23**	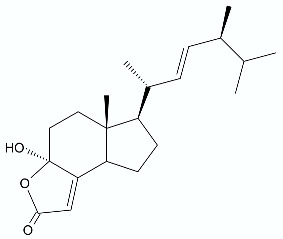 Dimethylincisterol A2	*B. subtilis*	10.26 ± 0.76 μg/mL	*Aspergillus hiratsukae* SCSIO 5Bn_1_003	[42]
**24**	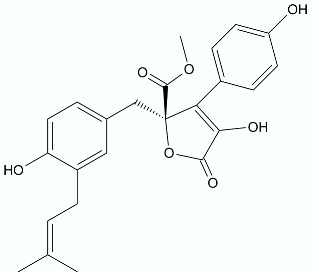	*S. aureus* *B. subtilis*	59.54 ± 0.50 μg/mL 5.30 ± 0.29 μg/mL	*Aspergillus hiratsukae* SCSIO 5Bn_1_003	[42]
**25**	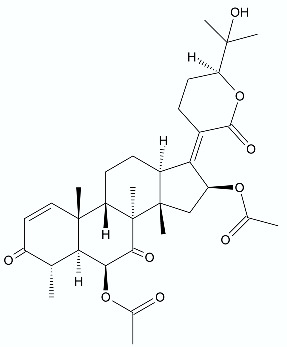	*S. agalactiae*	64 μg/mL	*Aspergillus fumigatus* HNMF0047	[43]
**26**	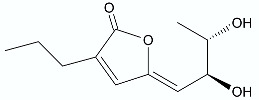	*S. aureus*	6.25 μg/mL	*Penicillium* sp. TGM112	[44]
**27**	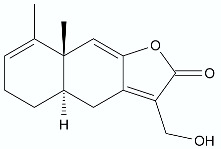	*E. coli* *B. subtilis* *S. aureus*	NI Inhibition diameter (Ø mm) 21 mm 22 mm 21 mm	*Eutypella* sp.	[45]
**28**	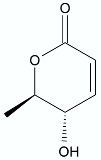	*A. baumannii* *E. coli*	10 μg/mL 10 μg/mL	*Tapinella atrotomentosa*	[47]
**29**	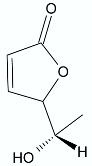	*A. baumannii* *E. coli*	6 μg/mL 10 μg/mL	*Tapinella atrotomentosa*	[47]
**30**	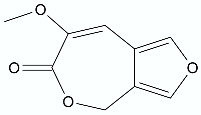 Penicillilactone A	*V. harveyi*	8 μg/mL	*Penicillium* sp. *LS54*	[48]
**31**	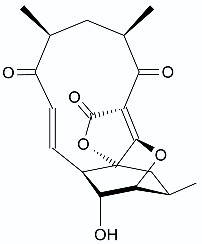 Abyssomicin C	*S. aureus*	4–13 g/mL	*actinomycete*	[22]
**32**	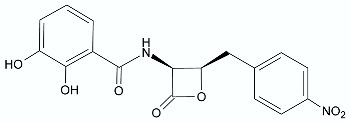 Obafluorin	*B. subtilis* *E. coli* ATCC 25922 *E. coli* ATCC 25922 pJH10TS *E. coli* NR698 *E. coli* NR698 pJH10TS	4 μg/mL 256 μg/mL 256 μg/mL 4 μg/mL 8 μg/mL	*Pseudomonas fluorescens* ATCC 39502	[50]

NI—MIC values not indicated.

## Data Availability

Not applicable.
